# Secondary hypertension in patients with saccular intracranial aneurysm disease: A population based study

**DOI:** 10.1371/journal.pone.0206432

**Published:** 2018-10-31

**Authors:** Satu Kotikoski, Jukka Huttunen, Terhi J. Huttunen, Katariina Helin, Juhana Frösen, Timo Koivisto, Mitja I. Kurki, Mikael von und zu Fraunberg, Ilkka Kunnamo, Juha E. Jääskeläinen, Antti E. Lindgren

**Affiliations:** 1 Neurosurgery of NeuroCenter, Kuopio University Hospital, and Institute of Clinical Medicine, School of Medicine, Faculty of Health Sciences, University of Eastern Finland, Kuopio, Finland; 2 The Finnish Medical Society Duodecim, Helsinki, Finland; 3 Department of Neurology and Neurosurgery, UMC Utrecht, Utrecht, the Netherlands; Universitatsklinikum Freiburg, GERMANY

## Abstract

**Background:**

Secondary hypertension is a serious form of hypertension, involving 5% to 10% of all hypertension patients. Hypertension is a risk factor of the saccular intracranial aneurysm (sIA) disease and subarachnoid hemorrhage from ruptured sIA (aSAH), but the impact of secondary hypertension on sIA disease is poorly known. In a defined Eastern Finnish sIA population we studied the prevalence of secondary hypertension and its impact on sIA disease phenotype.

**Methods:**

We included 2704 consecutive sIA patients first admitted to Kuopio University Hospital from 1995 to 2014. Their clinical data from Kuopio Intracranial Aneurysm patient and Family Database was fused with prescription drug usage data, hospital diagnoses and causes of death, retrieved from nationwide registries. Medical records of hypertensive sIA patients were reviewed to confirm or exclude secondary hypertension. Prevalence of secondary hypertension and associated diagnoses were calculated. Logistic regression was used to identify clinical characteristics of sIA disease that associated with secondary hypertension.

**Results:**

We identified 2029 (75%) sIA patients with hypertension and 208 (10%) of them had secondary hypertension. Most frequent conditions associated with secondary hypertension were kidney and renovascular diseases (45%), sleep apnea (27%) and hypothyroidism (19%); 46 (22%) of the 208 patients had more than one such condition. In multivariate logistic regression analyses of 1561 aSAH patients, secondary hypertension significantly associated with the number of sIAs (p = 0.003; OR 1.32; 95% CI 1.10–1.58) and male gender (p = 0.034; OR 1.59; 95% CI 1.04–2.43).

**Conclusions:**

Secodary hypertension was relatively common (10%) among hypertensive sIA patients. Secondary causes for hypertension should be taken into account in hypertensive sIA patients, especially in aSAH patients with multiple intracranial aneurysms. Further research is indicated to evaluate the impact of secondary hypertension on the long-term rupture risk of unruptured sIA carriers and long-term outcome after aSAH.

## Introduction

The prevalence of saccular intracranial aneurysm (sIA) disease in general population is around 3% [1]. Most sIAs do not cause symptoms and remain unnoticed during life [2], if not incidentally found in neuroimaging for other reasons or by screening sIA families [3]. Aneurysmal subarachnoid hemorrhage (aSAH), most often caused by rupture of sIA wall is a severe form of stroke with high mortality and neurological morbidity [4–10]. Known risk factors for sIA disease and aSAH include female gender, age, smoking, hypertension, sIA family, and autosomal polycystic kidney disease (ADPKD) [2, 9–14].

Secondary hypertension, resulting from an underlying identifiable cause, is a more serious form of hypertension, involving about 5% to 10% of all hypertension patients [15–18]. Etiologies for secondary hypertension include chronic kidney diseases [19], renovascular hypertension [20], primary aldosteronism [21], and obstructive sleep apnea [22], as well as number of rare causes [17]. Reaching the diagnosis of secondary hypertension is often challenging; indicators include young age, persistent hypertension requiring more than three types of anti-hypertensive medication, weakened response to previous medications, and exceptionally high blood pressure levels [23, 24]. Proper treatment of secondary hypertension may reduce the risk of serious cardiovascular complications, including heart disease, kidney failure and stroke.

Hypertension is a common risk factor of sIA disease and aSAH [2, 25], but published data on the occurrence and significance of secondary hypertension in sIA patients has remained scarce. The Kuopio Intracranial Aneurysm Patient and Family Database (www.kuopioneurosurgery.fi) contains all cases of unruptured and ruptured sIAs admitted to the Kuopio University Hospital (KUH) from a defined Eastern Finnish catchment population since 1980 [12]. The present study included 2704 sIA patients (1143 unruptured and 1561 ruptured) first admitted to KUH from 1995 to 2014. Their registry data, including the diagnoses, prescribed drugs and causes of deaths, were obtained from the nationwide registries and fused with the clinical data[2,7,8,13]. We retrospectively studied the sIA patients to define the prevalence of secondary hypertension on carriers of sIA disease and its impact on sIA disease phenotype.

## Materials and methods

### Study population

#### Kuopio Intracranial Aneurysm Patient and Family Database

During the study period from 1995 to 2014 KUH Neurosurgery exclusively provided neurosurgical services for a defined KUH catchment population in Eastern Finland. Geographical area remained the same but population decreased from 882671 to 840587. The median age increased from 37 to 42 in males and from 40 to 45 in females, and the proportion of males remained unchanged at 49% [8, 12].

All patients with verified SAH by CT or spinal tap have been admitted to KUH for angiography and treatment if not moribund. Unruptured sIAs have been verified by four-vessel digital subtraction angiography, magnetic resonance angiography or computed tomography angiography. Patients with unruptured IAs have also had neurosurgical consultation for the treatment and follow-up. KUH Neurosurgery maintains a database of all cases ruptured and unruptured intracranial aneurysms admitted to KUH since 1980. The database has been prospective since 1990 [12]. A dedicated full-time research nurse runs the database, interviews all new cases, and collects and codes detailed information into variables, including family history, defined as at least two affected first-degree relatives [12, 26].

**Fig 1:**
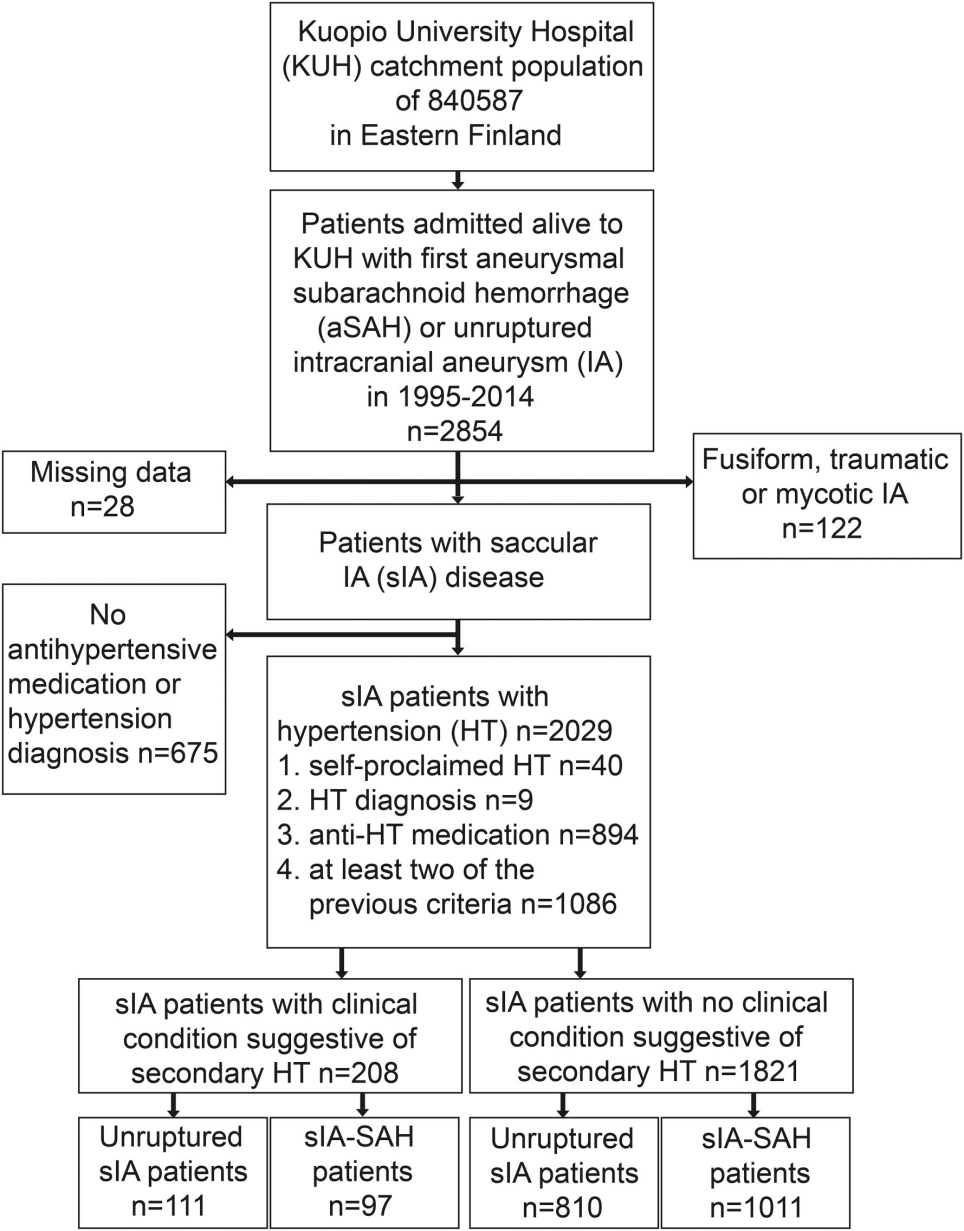
Flowchart of the study population: 2.704 patients with saccular intracranial aneurysm (sIA) disease admitted to the Kuopio University Hospital (KUH) with unruptured sIA disease or first subarachnoid hemorrhage (aSAH) from the Eastern Finnish catchment population from 1995 to 2014. Identification of the hypertensive sIA patients with comorbid diseases suggestive of secondary hypertension.

The basic study population included all sIA patients from 1995 to 2014 (Fig 1). The final study population consisted of unruptured and ruptured sIA patients who met the following criteria: 1) a citizen of Finland and resident of the KUH catchment area at the first diagnosis of the sIA disease from 1995 to 2014; 2) verification of the sIA disease with four-vessel angiography; 3) patients with other types of IAs (fusiform, traumatic, mycotic) excluded (Fig 1).

### Antihypertensive medication

The Social Insurance Institution of Finland has since 1994 prospectively maintained a nationwide registry for all patients who have purchased prescribed drugs from the pharmacies, including all antihypertensive drugs. All pharmacies in Finland are included in the prospective registry. In Finland all antihypertensive drugs are sold in pharmacies by prescription only. The antihypertensive medication use in the study population of 2704 sIA patients (Fig 1) was analyzed between January 1, 1995, and December 31, 2014, allowing us to analyze antihypertensive medication use at least one year before the sIA diagnosis. The data contained information since the first purchase date and the number of purchases until the last date. Purchases of antihypertensive drugs with ATC codes C02 (antihypertensives), C03 (diuretics; thiazides), C04 (peripheral vasodilators), C07 (beta blocking agents), C08 (calcium channel blockers), and C09 (agents acting on the renin-angiotensin system) were included in the analysis.

### Hospital diagnoses and death records

All hospital diagnoses (ICD-10), including hypertension diagnoses were obtained from the Finnish electronic hospital diagnosis registry (Care Register For Health Care HILMO, managed by the Finnish Institution for Health and Welfare) that covers all secondary and tertiary referral hospitals in Finland and encompasses all medical specialties. Death records and death diagnoses (ICD-10) were obtained from Statistics Finland. Hospital diagnoses and causes of death were then fused with the clinical data.

#### Diagnosis of hypertension

We defined hypertension was defined as one of the following: 1) self-proclaimed hypertension; 2) hypertension diagnosis by a physician; or 3) prescribed antihypertensive medications (Fig 1).

#### Diagnosis of secondary hypertension

Secondary hypertension was suspected in hypertensive sIA patients with one or more concomitant morbidities that associate with suspected secondary hypertension (Table 1), and their case reports were reviewed to confirm or exclude secondary hypertension as described in ESH/ESC (European Society of Cardiology http://www.escardio.org/Guidelines) guidelines [24].

**Table 1:** 208 sIA patients with one or more clinical conditions (n = 260) considered as etiological factors for their secondary hypertension among 2704 sIA patients admitted to Kuopio University Hospital from the Eastern Finnish catchment population in 1995–2014.

Clinical condition and ICD-10 classification	Patients n = 208
*Kidney and renovascular diseases*	
Polycystic kidney disease (Q61.2, Q61.3)	27 (13%)
Kidney failure (N17-19)	17 (8.2%)
Glomerulonephritis and nephrotic syndrome (N00-N08)	15 (7.2%)
Diabetic nephropathy (E10.2, E11.2)	9 (4.3%)
Hydronephrosis (N13.0, N13.1, N13.3, N13.9)	9 (4.3%)
Tubulo-interstitial nephritis and other conditions (N11-12, N14-16)	6 (2.9%)
Malignant neoplasm of kidney (C64)	6 (2.9%)
Atherosclerosis of renal artery (I15.0, I70.1)	4 (1.9%)
Fibromuscular dysplasia (I77.3)	1 (0.5%)
Coarctation of the aorta (Q25.1)	2 (1.0%)
*Adrenal gland diseases*	
Benign or malignant neoplasm of adrenal gland (D35.0, C74)	6 (2.9%)
Hyperaldosteronism (E26)	2 (1.0%)
*Other hormonal diseases*	
Hypothyroidism (E03)	39 (19%)
Hyperthyroidism (E05)	14 (6.7%)
Hyperparathyroidism (E21.0-E21.3)	10 (4.8%)
Benign neoplasm of pituitary gland (D35.2)	6 (2.9%)
Acromegaly (E22.0)	2 (1.0%)
*Systemic diseases*	
Sjögren’s syndrome (M35.0)	5 (2.4%)
Familial dysautonomia (Riley–Day syndrome) (G90.1)	5 (2.4%)
Systemic lupus erythematosus (M32)	5 (2.4%)
Other specified or unspecified necrotizing vasculopathy (M31.8, M31.9)	3 (1.4%)
Amyloidosis (E85) with kidney failure or nephritic syndrome	2 (1.0%)
Thrombotic microangiopathy (M31.1)	2 (1.0%)
Giant cell arteritis (M31.5)	2 (1.0%)
Henoch-Schönlein purpura (D69.0)	1 (0.5%)
Rheumatoid vasculitis with rheumatoid arthritis (M05.2)	1 (0.5%)
Polyarteritis nodosa (M30.0)	1 (0.5%)
Wegener’s granulomatosis (M31.3)	1 (0.5%)
Systemic sclerosis (M34)	1 (0.5%)
Sleep apnea (G47.3)	57 (27%)

### Ethical aspects

This research has been authorized by Kuopio University Hospital Reseach Ethics Committee. The patient data integration from the nationwide registries to Kuopio IA database was completed with the endorsement from National Institute of Health and Welfare. Written informed consent was obtained from all patients before their data was input to Kuopio Intracranial Patient and Family database.

### Statistical analysis

Categorical variables were expressed in proportions and continuous variables in medians, quartiles, and ranges. Groups were compared using the Pearson’s Chi-Square (χ2) test or the Mann–Whitney U test. Multivariate analyses were performed using logistic regression to calculate odds ratios (ORs) with corresponding 95% confidence intervals (CIs) for having secondary hypertension with gender, age at first diagnosis, familial sIA disease and number sIAs as variables. P values <0.05 were considered significant. SPSS 22 statistical software was used (SPSS, Inc, Chicago, IL).

## Results

The study population consisted of 2704 sIA patients, 1143 (42%) with unruptured sIA disease and 1561 (58%) with aSAH, admitted to KUH between 1995 and 2014 (Fig 1). Their follow-up ended at death (n = 776) or December 2014, with total follow-up of 18751 patient-years and a median follow-up time of 68 months. Table 2 presents the characteristics of the 2704 sIA patients and their 3922 sIAs.

**Table 2:** Secondary vs. essential hypertension among 2704 patients with 3922 saccular intracranial aneurysms (sIAs) admitted to Kuopio University Hospital from the Eastern Finnish catchment population in 1995–2014.

	Unruptured sIA patients n = 1143			aSAH patients n = 1561		
Variables of 2704 sIA patients	Hypertension		No hypertension n = 222	Hypertension		No hypertension n = 453
	Secondary hypertension n = 111	No secondary hypertension n = 810		Secondary hypertension n = 97	No secondary hypertension n = 1011	
Median age at sIA diagnosis (quartiles)	58 (51–65)	59 (50–68)	50 (42–57)	55 (46–65)	55 (47–65)	50 (42–60)
Females	64 (58%)	475 (59%)	120 (54%)	48 (50%)	624 (62%)	236 (52%)
Familial sIA disease	18 (16%)	143 (18%)	49 (22%)	16 (16%)	105 (10%)	36 (8%)
Multiple sIAs (≥2)	32 (29%)	236 (29%)	56 (25%)	35 (36%)	310 (31%)	117 (26%)
Known positive smoking history	40 (36%)	351 (43%)	108 (49%)	48 (49%)	427 (42%)	193 (43%)
Variables of 3922 sIAs	Hypertension		No hypertension n = 303	Hypertension		No hypertension n = 639
	Secondary hypertension n = 160	No secondary hypertension n = 1148		Secondary hypertension n = 170	No secondary hypertension n = 1502	
Median size (mm) (quartiles)	4 (3–7)	4 (3–7)	4 (3–6)	4 (3–7)	5 (3–8)	6 (3–8)
ACoA location	25 (16%)	166 (14%)	34 (11%)	39 (23%)	376 (25%)	148 (23%)
Mbif location	55 (34%)	442 (39%)	119 (39%)	58 (34%)	433 (29%)	175 (27%)
ICA location	32 (20%)	263 (23%)	88 (29%)	38 (22%)	321 (21%)	164 (26%)
BAbif location	6 (4%)	62 (5%)	10 (3%)	8 (5%)	71 (5%)	24 (4%)
Other location	42 (26%)	215 (19%)	52 (17%)	27 (16%)	301 (20%)	128 (20%)
Irregular shape	33 (21%)	271 (24%)	83 (27%)	87 (51%)	932 (62%)	417 (65%)
Smooth shape	126 (79%)	861 (75%)	210 (69%)	80 (47%)	525 (35%)	188 (29%)
Unknown shape	1 (1%)	16 (1%)	10 (3%)	3 (2%)	45 (3%)	34 (5%)

Abbreviations: sIA = saccular intracranial aneurysm; aSAH = subarachnoid hemorrhage from ruptured sIA; ACoA = anterior communicating artery; Mbif = middle cerebral artery bifurcation; ICA = internal carotid artery; BAbif = basilar artery bifurcation.

Of the study population, 2029 (75%) sIA patients had hypertension (Fig 1, Table 2) and 208 (10%) of them had secondary hypertension (Table 1). Of the patients with secondary hypertension, 46 (22%) had more than one condition associated with secondary hypertension. The most frequent conditions associated with secondary hypertension were kidney and renovascular diseases (45%), sleep apnea (27%) and hypothyroidism (19%) (Table 1). Of the 208 patients with secondary hypertension 88 (42%) had a positive smoking history.

In the 111 unruptured sIA patients, the median ages (25% and 75% quartiles) at diagnoses were 50 (43–58) years for hypertension, 54 (42–62) years for secondary hypertension causing condition and 58 (51–65) years for unruptured sIA disease. In the 97 aSAH patients, the median ages (25% and 75% quartiles) at diagnoses were 50 (43–61) years for hypertension, 52 (40–67) years for secondary hypertension causing condition and 55 (46–65) years for aSAH.

In total, we identified 26 (13%) carriers of autosomal dominant polycystic kidney disease (ADPKD), while other heritable traits predisposing to hypertension were rare: 5 (2.4%) patients with familial dysautonomia (Riley–Day syndrome).

Of the 367 familial sIA patients, 282 (77%) had the hypertension diagnosis and 34/282 (12%) had secondary hypertension. Of the 2337 sporadic sIA patients, 1747 (75%) had hypertension diagnosis and 174/1747 (10%) had secondary hypertension.

In multivariate logistic regression analyses of aSAH 1561 patients, secondary hypertension significantly associated with the number of sIAs (p = 0.003; OR 1.32; 95% CI 1.10–1.58) and male gender (p = 0.034; OR 1.59; 95% CI 1.04–2.43) (Table 3). For the 1143 unruptured sIA patients, no significant associations with secondary hypertension were found.

**Table 3:** Multivariate logistic regression analysis of factors associated with secondary hypertension in the 1561 patients with subarachnoid hemorrhage from ruptured saccular intracranial aneurysm (aSAH) admitted to the Kuopio University Hospital from the Eastern Finnish catchment population from 1995 to 2014.

	OR (95% CI)	p-value
Male gender	1.59 (1.04–2.43)	0.034
Age at aSAH (per year)	1.01 (0.99–1.03)	0.33
Familial sIA disease	1.74 (0.98–3.09)	0.058
Number of sIAs (per sIA)	1.32 (1.10–1.58)	0.003

## Discussion

In this population-based retrospective study of 2704 sIA patients, we showed that 75% had hypertension and 10% of them were considered to have secondary hypertension, with renovascular diseases, sleep apnea and hypothyroidism as most common associated disorders. Interestingly, secondary hypertension significantly associated with the number of sIAs in aSAH patients.

In general Finnish population, the prevalence of secondary hypertension among all hypertension patients has beeb estimated to be 5–10% in the Finnish national hypertension treatment guideline [27], in line with our results. This estimate is based on two Swedish studies [15–16]: The prevalence of secondary hypertension was 5.8% in a prospective random population sample of 7455 Swedish men [15] and 4.7% in a retrospective study of 1000 hypertension patients sent to a hypertension clinic due to treatment-resistant or newly diagnosed hypertension [16]. In a prospective Japanese study from 2003, the prevalence of secondary hypertension was 9.1% in 1020 hypertensive general outpatient clinic visitors [17]. Possible etiological conditions included aldosteronism (6%), Cushing’s syndrome (1%), preclinical Cushing’s syndrome (1%), pheochromocytoma (0.6%) and renovascular hypertension (0.5%). Importantly, the patients with unspecified renal failure were excluded from the study and no data on sleep apnea or hypothyroidism was given. Hypothyroidism and sleep apnea were frequent in secondary hypertension patients with IAs in our population, which is in line the prevalence estimates of 3,1% [28] for hypothyroidism in and 3,8% [29] for sleep apnea in general European population.

Our study has several strengths derived from the Finnish health care system. Firstly, Finland is divided into mutually exclusive catchment areas for the KUH and the four other university hospitals, allowing cohorts that are unselected and minimally biased. Finnish personal identification code system allows the creation of very accurate population and clinical data registries [30] and the linking of the registry data with medical records. Secondly, during the study period (1995–2014), the Kuopio Intracranial Aneurysm and Family Database prospectively collected clinical data according to a standard protocol of all unruptured and ruptured sIA patients since their first admission from the KUH catchment population. All sIAs have been verified with four-vessel angiography, excluding the patients with other types of IAs (traumatic, fusiform and mycotic). Thirdly, in Finland, all anti-hypertensive medications are exclusively sold by the physicians’ prescriptions in the pharmacies, and all prescribed drug purchases were retrieved from the national registry encompassing all pharmacies in Finland. Furthermore, the study period 1995–2014 allowed the assessment of prescription drug usage at least one year before sIA diagnosis.

Our study also has weaknesses. Firstly, our study is a retrospective registry-based study. We did not invite patients for clinical visits to be screened for secondary hypertension. The method we used to diagnose secondary hypertension may be insensitive even though it was based on thorough review medical records and combination of comprehensive nationwide registries. It is likely that we have missed patients with secondary hypertension and the actual prevalence may be even higher. Secondly, in some cases the diagnoses associated with secondary hypertension may not have been causes for hypertension, but coincidental findings. Furthermore, due to the method used, we were not able to determine the severity of hypertension. However, we feel that our large study population analyzed with a consistent method to identify patients with secondary hypertension compensates for this limitation. Moreover, identifying definite causative relations in secondary hypertension is difficult regardless of the method used.

### Conclusion

In conclusion, we found that secondary hypertension is a relatively common disease in patients with sIA disease, with 10% prevalence in hypertensive sIA carriers. In clinical practice, secondary hypertension may be an overlooked risk factor in patients with subarachnoid hemorrhage. Our results indicate that secondary causes for hypertension should be taken into account in hypertensive sIA patients, especially in aSAH patients with multiple intracranial aneurysms. In these patients, screening for kidney and thyroid disorders and sleep apnea should be considered if hypertension is diagnosed. Further research is indicated to evaluate the impact of secondary hypertension on the long-term rupture risk of unruptured sIA carriers and long-term outcome after subarachnoid hemorrhage.
